# Comparing the rules of engagement of androgen and glucocorticoid receptors

**DOI:** 10.1007/s00018-017-2467-3

**Published:** 2017-02-06

**Authors:** Frank Claessens, Steven Joniau, Christine Helsen

**Affiliations:** 1 Molecular Endocrinology Laboratory, Department of Cellular and Molecular Medicine, KU Leuven, Campus GHB ON1, Herestraat 49, PO Box 901, 3000 Leuven, Belgium; 2 Department of Urology, University Hospitals Leuven, Herestraat 49, 3000 Leuven, Belgium

**Keywords:** Androgen receptor, Glucocorticoid receptor, DNA binding, DNA-response element, Prostate Cancer

## Abstract

Despite the diverse physiological activities of androgens and glucocorticoids, the corresponding receptors are very close members of the nuclear-receptor super family. Their action mechanisms show striking similarities, since both receptors recognize very similar DNA-response elements and recruit the same coactivators to their target genes. The specificity of the responses lies mainly in the tissue-specific expression of the receptors and in their ligand specificity. In cells, where both receptors are expressed, the mechanisms leading to the difference in target genes are less obvious. They lie in part in subtle variations of the DNA-binding sites, in cooperativity with other transcription factors and in differential allosteric signals from the DNA and ligand to other receptor domains. We will highlight the different suggestions that might explain the DNA sequence selectivity and will compare the possible allosteric routes between the response elements and the different functions in the transactivation process. The interplay of androgen and glucocorticoid receptors is also highly relevant in clinical settings, where both receptors are therapeutically targeted. We will discuss the possibility that the glucocorticoid and androgen receptors can play partially redundant roles in castration-resistant prostate cancer.

## Introduction

The physiological roles of androgens and glucocorticoids are very different, and this is reflected in their clinical applications. Androgens are the male sex steroids mainly involved in development and maintenance of reproductive organs and spermatogenesis. Clinically, they might provide benefit as hormone replacement therapy for hypogonadal men or in cases of severe cachexia or osteoporosis [1, 2]. On the other hand, in the therapy of metastatic castration-resistant prostate cancer, androgen action is blocked by androgen deprivation, androgen synthesis inhibitors, and/or the use of androgen receptor (AR) antagonists [3]. Glucocorticoids, however, are mainly controlling inflammation and metabolism which has led to their wide-spread use to treat inflammatory and immunologic disorders [4].

**Fig. 1:**
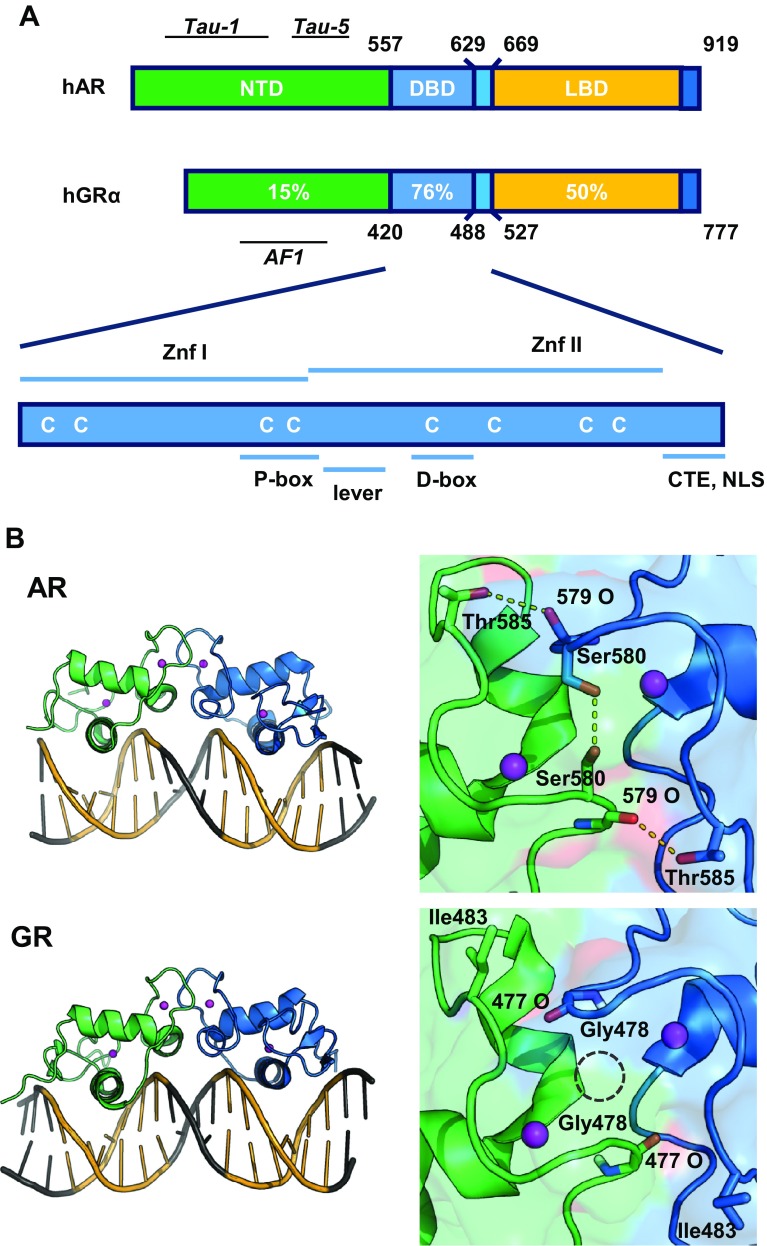
**a** Comparison of the domain structure of the androgen and glucocorticoid receptor (AR and GR), with indication of the different domains and level of conservation. *NTD* amino-terminal domain, *DBD* DNA-binding domain, *LBD* ligand-binding domain; *Znf* Zinc finger, *CTE* carboxyterminal extension, *NLS* nuclear localization signal. **b** Structure of the DNA-binding domains of the AR and GR bound to DNA (*left* panels). At the *right*, details of the D-boxes with dotted lines indicating AR-specific H-bonds and the GR specific glycine hole (adapted from [12])

Androgen and glucocorticoid effects are mediated by their corresponding receptors, the AR and the glucocorticoid receptor (GR), which are both nuclear receptors. Nuclear receptors are ligand-inducible transcription factors; they have a centrally located DNA-binding domain (DBD) which is connected via a hinge region to a carboxyterminal ligand-binding domain (LBD) and an amino-terminal activation function (NTD) (Fig. 1a). The DBD exists of two zinc-coordinating modules and forms the signature domain of the nuclear-receptor family. Because of their essential roles in target gene selection, both DBD and hinge region as well as the response elements will be discussed in detail.

The LBDs of all nuclear receptors are folded in a typical 3-layered, 12-helical structure with a ligand-binding cavity [5]. While the overall structure of the LBD is conserved, receptor-specific residues delineating the ligand-binding cavity determine the ligand specificity of the receptors. The binding of the cognate ligand is proposed to induce a repositioning of the carboxyterminal helix (H12) of the LBD, thus forming an activation function (called AF2) which is a docking site for α-helical LxxLL motifs that are found in most coactivator complexes [6].

The NTD of the nuclear receptors is very diverse both in length and sequence. In addition, between the NTDs of AR and GR, there is little or no conservation, although both have transcription activating properties (called AF1). A comparison between the NTD of AR and GR has been extensively reviewed elsewhere [7–9].

Here, we will compare how the AR and GR interact with DNA and chromatin; how they evoke tissue-specific transcriptional responses and in how far they have interchangeable functions, for instance, in prostate cancer [10, 11].

## Comparing the DNA-binding domains of AR and GR

AR and GR are steroid receptors which form a specific subfamily of the nuclear receptors. Steroid receptors can be divided based on their sequence specificity into two groups: the estrogen receptors (ER) and ER-like receptors on one hand and the glucocorticoid, mineralocorticoid (MR), progesterone (PR), and androgen receptors on the other hand. The latter are also called the oxosteroid receptors.

The structures of the DNA-binding domains of nuclear receptors were solved by X-ray crystallography [12, 13] and later refined by NMR [14]. The DNA-binding domains of all steroid receptors are very similar (Fig. 1a). Part of the first zinc finger (P-box) folds into an α-helix which fits in the major groove of the DNA thus making sequence-specific contacts with 5′-AGAACA-3′-like or 5′-GGTACA-3′-like motifs [15]. Part of the second zinc finger (D-box) is involved in receptor dimerization. Because of this specific D-box dimerization, the DNA-recognition helices of the two DBDs are positioned at a very specific distance relative to one another. This structural characteristic determines that all steroid receptors recognize bipartite DNA elements with exactly three nucleotide spacers, called inverted repeat with 3-nucleotide spacer (IR3). There is an important, strong cooperativity between the two monomers when they bind to DNA [15].

A detailed comparison of the DBDs from AR and GR reveals many structural similarities, but also some remarkable differences. The amino acids in the DNA-binding helix, which make the sequence-specific contacts with the bases, are identical in GR and AR, but small nuances in the three-dimensional structure of the α-helix indicate a slightly stronger affinity of the AR-DBD for its 5′-AGAACA-3′ motif [16]. For the AR-DBD, there is only one crystal structure available (Fig. 1b), but for the GR-DBD, a series of NMR structures with different GREs and receptor isoforms were reported [17, 18]. This revealed a bidirectional allosteric signaling role for the so-called ‘lever arm’, transmitting signals from the DNA reading head and the DBD dimerization surface to other functional domains of the receptor. As a consequence, GREs that differ in one base only differentially affect GR transactivating properties [17]. It is interesting to note that the sequence of the lever arm is different between GR and AR which implies different receptor-specific allosteric pathways.

**Fig. 2:**
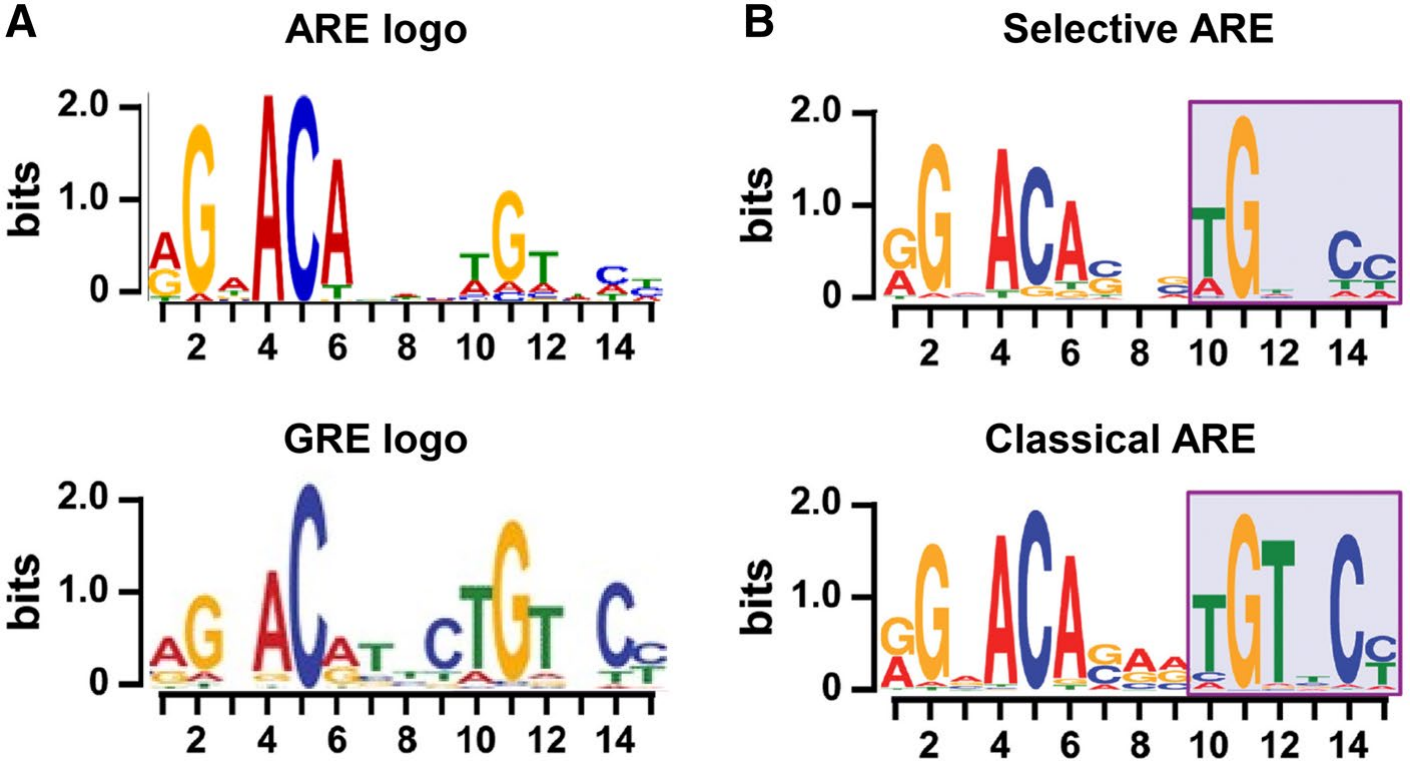
**a** Logo presentation of top-enriched ARE motifs provided by Cheung based on [99] (*top*) and top-enriched GRE motifs from [35] (*bottom*). **b** Logo presentation of top-enriched ARE motifs specific for the SPARKI model, considered to be selAREs (*top*) or for classical AREs (*bottom*). Adapted from [48]

The amino acids of the second zinc finger which constitute the dimerization surface of the DBDs are also conserved between the oxosteroid receptors (Fig. 2b). Nevertheless, there are two AR-specific residues, Ser598 (Gly in GR, PR, and MR) and Thr603 (Ile in GR, PR, and MR) that contribute to the stronger DNA-dependent dimerization for AR. The Ser598 of AR not only increases the van der Waals forces, but also adds two hydrogen bonds to the interface. In case of a glycine at this position, a molecular cavity is apparent between the two DBDs (Fig. 1b). Moreover, Thr603 in the AR-DBD can form additional hydrogen bonds which cannot be formed by the corresponding isoleucine in the GR, PR, or MR (Fig. 1b) [12, 16].

In conclusion, although the two zinc fingers of the AR and GR-DBDs differ only in 12 out of 65 residues and even when most of the substitutions are conservative, small variations in structure seem to have functional consequences. These subtle differences in the way steroid receptors bind DNA correlated with slightly different consensus binding sites and overlapping but distinct sequence specificities between oxosteroid receptors, which will be discussed further [19, 20]. Surprisingly, the differences in P-box sequence between AR and ER, which control a different set of target genes in vivo, are insufficient to impose gene selectivity *in vitro*. Indeed, the P-box of the ER-DBD makes sequence-specific contacts with the 5′-AGGTCA-3′ hexamer half sites of the estrogen response elements (ERE), while the oxosteroid P-box recognizes their cognate 5′-AGAACA-3′ hexamer. However, despite these differences, the AR is able to bind EREs and this has been proposed to be a mechanisms by which it interferes with ER functioning in breast cancer [21]. Observations like these clearly demonstrate that the DNA binding as such cannot explain the specificity of the physiological steroid responses. What the role of the other receptor domains could be during the selection of the target enhancers is being discussed in the following sections.

## Comparing androgen and glucocorticoid response elements

The lack of receptor specificity of the first identified response elements for glucocorticoids, progestagens, mineralocorticoids, and androgens has been confusing. Indeed, in binding and transactivation assays, all glucocorticoid response elements (GRE) are recognized by AR, MR, and PR and vice versa; many androgen response elements (AREs) are recognized by the other oxosteroid receptors [22–24]. In addition, in more recent chromatin immunoprecipitation assays (ChIP-seq), the in silico derived consensus sequences for AR and GR binding motifs were quite similar (Fig. 2a). How can receptors with such diverse physiological roles act through DNA elements with so similar sequences? A large part of the answer must lie in the cell-specific chromatin environment [25]. For GR, for example, only a 5% overlap was seen between its binding sites in mammary versus pituitary cell types indicating a crucial role for epigenetic chromatin factors in intracellular receptor-DNA binding [26]. Cell-specific transcription factor occupancy of enhancers and promotors can also contribute to receptor selectivity. An example for this is the pioneering role of FoxA1 which specifies AR DNA binding in LNCaP cells [27]. However, what happens when cells express both AR and GR and when both ligands are present? In LNCaP cells that express both AR (endogenously) and GR (exogenously), or VCaP cells that express both receptors (endogenously), there is a large overlap between AR and GR binding sites indicating that the receptors might bind the same DNA elements [27, 28]. These common binding sites are located in active enhancers near genes that are responsive to both hormones. Because the activity of some of these genes is correlated with oncogenic processes, this supports the hypothesis that re-expression of GR in castration-resistant prostate cancer could explain the progression into an AR-independent form of the disease [10, 11].

### AR and GR both bind to the AGAACA consensus sequence organized as an inverted repeat with a 3n spacer

The first systematic discovery of genomic AR binding sites was based on ChIP-on-chip and ChIP-seq experiments on AR-positive prostate cancer cell lines. While the earlier, limited ‘old school’ biochemical analyses indicated that AR binds DNA motifs that are organized as 5′-AGAACA-3′-like repeats, initial *in silico* analyses of the ARBS lead to the idea that the AR might also bind monomeric motifs or dimeric motifs with variable spacing and orientations [29–31]. However, later mutational analyses of such putative alternative AREs strongly indicated that the AREs are always dimeric in nature with an exact 3 nucleotide spacer [24, 32]. A study on DNA specificity of human transcription factors that used high-throughput SELEX for determining binding sites also pointed out the dimeric nature of the binding motif of the AR with 5′ GTACA 3′ as the consensus half site [33]. Chen et al. described in another study that the sequence specificity of the AR depended on the ligand. In LNCaP cells, agonist bound AR binds the classical inverted repeat-like elements, while in the genomic binding sites for antagonist-bound AR, elements which resemble a 5′-CnnG-3′ repeat with a 5 nucleotide spacer are enriched [34].

While the data for monomeric binding are less convincing for AR, monomeric binding for the GR has been reported to some of its enhancers [35]. Moreover, ChIP-exo data, which give a more detailed indication of the exact borders of the receptor-binding motifs, revealed mainly dimeric binding sites for GR and a redistribution to monomeric sites when the D-box of the GR is mutated [36, 37].

It should be noted that in this review, we do not discuss the possibility of indirect DNA binding, which has been well documented, certainly in case of the GR. Indeed, such GR tethering to DNA via other transcription factors could involve monomeric receptors and will result in receptor-specific effects on gene activation and/or repression [38–40]. In how far such monomeric GR might play a role in castration-resistant prostate cancer has not been resolved yet.

Despite the high similarity between AREs and GREs (Fig. 2a), we and others identified differential receptor recognition that could offer an alternative explanation for receptor specificity. A subset of AREs turns out not to be recognized by GR. When cloned upstream of a reporter gene, they confer responsiveness to androgens and progestins but not to mineralocorticoids or glucocorticoids [12, 41]. In vitro DNA-binding assays showed that AR and PR, but not MR or GR, bind these selective AREs (selARE) with high affinity. Moreover, the isolated GR-DBD binds these selAREs as monomers or as non-cooperative dimers [42, 43]. So what makes an ARE selective for AR?

### Discovery of selective AREs and the proposed differential AR binding mode

A comparison of the sequences of the first selAREs with that of the first known classical AREs led us to propose that the selAREs could be organized as direct repeats, rather than inverted repeats of 5′-AGAACA-3′-like hexamers [22, 24]. This was further corroborated by the observation that any synthetic direct repeat was able to confer androgen but not glucocorticoid responsiveness to reporter genes. Moreover, when mutations reduce the direct repeat-like nature of selective AREs, they gained responsiveness to glucocorticoids [19]. Does this mean that selAREs are bound by AR dimers in a head-to-tail conformation, much like many of the non-steroid receptors? [44]. This possibility was suggested by the fact that swapping of the dimerization interface between the DBDs of AR and GR also swapped the selectivity: an AR-DBD with the second zinc finger module of the GR no longer binds selective AREs, but still binds classical AREs [43, 45]. Vice versa, a GR-DBD with the second zinc finger of the AR gains affinity for selective AREs. However, the crystal structure of the AR-DBD on a direct repeat element shows a clear inverted protein dimer (head-to-head conformation) [12]. Therefore, unlike what has been reported for several other nuclear receptors, direct repeat binding does not mean head-to-tail dimerization in case of the AR [46].

To answer the question how common selective AREs are and whether this type of selectivity has any in vivo relevance, we developed the SPARKI mouse model. In this model, the exon encoding the second zinc finger of the AR was replaced for the corresponding exon of the GR gene. The resulting mutant AR can still bind to classical AREs, but no longer binds to or activates via selAREs; hence the name specificity affecting AR knockin (SPARKI) [45]. The SPARKI male mice develop a phenotype resembling partial androgen insensitivity [47]. Loss of binding to selAREs was first demonstrated by the loss of responsiveness in testes and epididymis of a subset of the androgen-regulated genes that subsequently were shown to have selAREs in their enhancers/promoters [45, 47]. More recent AR ChIP-seq on prostate and epididymis of SPARKI versus wild (WT) type mice also revealed distinctive patterns of chromatin binding by the WT-AR versus the SPARKI-AR [48]. While in wild-type epididymal tissue, 10 009 ARBS were picked up, only 6446 binding events were detected in the SPARKI tissue. This loss of AR binding events again correlated well with loss of androgen responsiveness of nearby genes as demonstrated in transcriptome analysis. Moreover, most of the SPARKI ARBS were also bound by AR in wild-type tissue and correlated with the vicinity of androgen-responsive genes, indicating that the SPARKI mutation led to the loss of a specific function of the AR and not in the complete inactivation of AR DNA binding.

The evaluation of the AR binding sequences in SPARKI and WT tissues allowed for the construction of a consensus sequence for selAREs (AR binding lost in SPARKI) and for classical AREs (AR binding retained in SPARKI and present in WT) (Fig. 2b). These two consensus sequences were similar but distinct [48]. The classical ARE consensus is virtually identical to the GRE consensus taken from Schiller [35]. The consensus of the selective elements is also very similar, except for a loss of thymine conservation at position 12 (Fig. 2b). Clearly, these similarities left little room for the hypothesis that selective AREs have a direct repeat nature.

Therefore, how can we explain the variation in sequence preference between AR and GR? For both receptors, it was postulated that the affinity for the upstream, more conserved 5′-AGAACA-3′ hexamer is high and binding to this hexamer results in a DNA-dependent dimerization with a second monomer that will bind hexamers that can diverge more from 5′-AGAACA-3′ [14–16]. It is tempting to speculate that the dimerization interface in the second zinc finger, which is stronger for the AR (Fig. 1b), allows more divergent downstream hexamers. This hypothesis is not confirmed by mutational analyses in which the Serine and Threonine in AR were swapped for the GR residues and vice versa, and the Glycine and Isoleucine in the GR were swapped for the AR residues in the GR [49].

Unfortunately, in the only crystal structure of the AR-DBD bound to DNA that has been published until now [12], the motif is organized as a direct repeat (based on the earlier report) with an adenine at position 12. Therefore, the final explanation for the lower stringency of the AR versus the GR or for the enrichment of the thymine at this position remains to be elucidated. Possibly, the difference in sequence specificity could be explained by alternative DNA or chromatin interactions contributed by the CTE or by receptor-specific allosteric interactions with other receptor domains or coactivators. This will be discussed in the following.

## Hinge region

Despite their name, hinge regions are more than the flexible connection between the DBDs and the LBDs. For the AR-, PR-, GR-, and ER-DBDs, it is clear that residues immediately carboxyterminal of the second zinc finger are involved in DNA binding (Fig. 1a) [50, 51]. Indeed, the addition of the CTEs to the DBD increased the binding affinity for the DNA-response elements [43]. While sequence specificity of the full size receptors is not affected by mutations or deletions in the CTE, clearly, the affinity for DNA was [52, 53]. For other nuclear receptors, the interactions of similar carboxyterminal extensions with the DNA immediately adjacent to the hexamers have been well documented [54]. For AR and GR, however, the possible contacts with the DNA still need to be elucidated because of the unstructured nature of the CTE in the thus far known crystals. Moreover, for both AR and GR, the CTE coincides with a nuclear localization signal known to interact with importin-α [55]. This is not the only function of the CTE, since the AR (629)RKLKK(633) motif was shown to be involved in intranuclear mobility, control of the N/C interactions (see in the following), and even the transactivation properties of the AR [52, 53]. Of course, these different functions are executed at different timepoints and at different locations in the cell involving different interaction partners. As an additional layer of complexity, these functions seem to be differentially affected by posttranslational modifications, such as the acetylation of the Lysines of the (629)RKLKK(633) motif [56–58].

## Allosteric signaling between the DNA elements and the other receptor domains

Like for most proteins, allostery plays an integrative role in the functioning of nuclear receptors. For the ligand-binding domains of NR, there are clear allosteric pathways identified between the ligand-binding pockets and the activation functions, where coregulators interact [59]. Many data also indicate allosteric communications between the DNA-binding domain and the DNA elements (reviewed in [60]). While intra-domain allostery within the DBD and LBD has been examined in great detail, the allostery between different domains is much harder to study. The best studied is the allostery from the GR-DBD, where the so-called lever arm situated between the two zinc finger modules plays a clear role in the transmission of sequence-specific signals from the DNA to coactivator recruitment by the LBD [17, 61]. This is further illustrated by the effect of a splice variant of the GR which has a single amino acid added to the lever arm and which dramatically affects the differential coregulator recruitment to the GR [18].

**Fig. 3:**
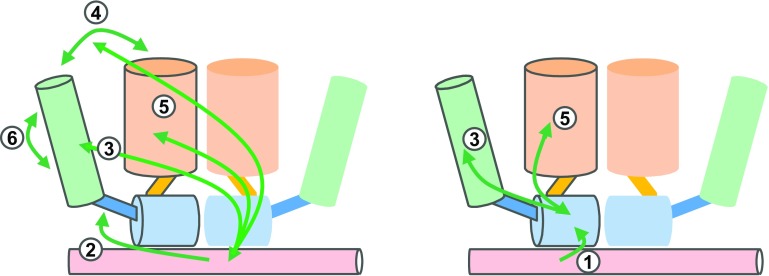
Schematic presentation of the allosteric signaling pathways between different domains of the AR or GR. Color code: *light red* DNA; *light blue* DBD; *blue* hinge; *green* NTD; *orange* LBD. *1* Signaling can take place between the DNA to the DNA-binding domain [17]; *2* between the DNA to the hinge [53]; *3* from the DNA to the amino-terminal domain [52, 63]; *4* from the DNA to the N/C interactions [63]; *5* from the DNA-binding domain to the ligand-binding domain [100]; and *6* between Tau-1 and Tau-5 [101]. For reasons of simplification, only allosteric pathways within one monomer are shown

Physiological evidence for allosteric effects in the AR comes from the functional analysis of AR-NTD mutations, and detected in androgen insensitivity patients that affect the functioning of AR in an ARE-specific way [62]. In addition, the deletion of the (23)FQNLF(27) motif which is involved in NTD-LBD communications in the AR has an effect on transactivation via classical AREs, but has little effect on reporters based on selAREs [63]. Moreover, the deletion of the polyglutamine tract or mutations in the sumoylation sites in the NTD have an effect on AR transactivation via classical elements, but not on selAREs [64]. Clearly, we need more detailed three-dimensional structures of the full-length androgen or glucocorticoid receptors, such as the ones described for other NRs [46, 65, 66]. Possible allosteric pathways between different domains for which there is some experimental, albeit sometimes circumstantial evidence are given in Fig. 3.

## Role of lncRNA in receptor functioning

The recent discovery of many thousands long noncoding RNA-encoding genes in the human genome will affect the study of virtually all biological processes [67]. These lncRNAs bind to protein-like transcription factors and coactivators and have been proposed to act as complex-building scaffolds. How many of them interact with nuclear receptors in general and steroid receptors more specifically, for example, remains to be determined. However, even before the term was coined, lncRNAs were known to interact functionally with the AR-NTD. This was first demonstrated for the steroid receptor RNA activator (SRA) by the group of O’Malley [68]. The structure–function analyses of SRA as well as the interacting proteins are still being identified [69, 70]. More recently, other lncRNAs, such as PRNCR1 and PCGEM1, have been proposed to affect AR functioning via its amino-terminal domain [71].

Another action mechanism was reported for ‘growth arrest-specific transcript’ called gas5. GR was shown to bind to a motif in this lncRNA, and this was proposed to squelch the receptor away from the genome [72]. The receptor-binding RNA motif in gas5 is a double-stranded fragment which resembles a 5′-AGAACA-3′-like monomeric DNA motif, hence called GRE mimic. *In vitro*, this RNA motif can titrate MR, PR, and AR activity in cellular overexpression experiments [73]. It will be exciting to learn how gas5 is implicated in the action mechanisms of these steroid receptors in normal physiology and in disease mechanisms. Because of the possible role of the NTDs, it is likely that here too, receptor specific mechanisms will exist.

In conclusion, over the coming years, we can expect a dramatic increase in our insights on how lncRNAs affect NR biology in many different physiological processes and disease mechanisms.

## Cooperativity with other transcription factors

The first AREs in eukaryotic genes were described for the androgen-dependent, prostate-specific C3 component of the prostatic binding protein [74, 75], the prostate-specific antigen [76], the probasin [77], and sex-limited protein genes [78]. For all these, it was immediately apparent that the AREs were part of complex enhancers to which several other transcription factors, such as NF1, Oct1, SP1, and GATA, need to bind to result in their full activity [78–82]. Indeed, mutation analyses of the individual binding sites within these complex enhancers illustrated the cooperative nature of the binding of transcription factors. Clearly, the AR is the ligand-induced factor which turns on the enhancers, but they themselves also depend on the binding of other enabling transcription factors to induce transcription of a nearby gene. ChIP-seq data on series of transcription factors in cell lines and more recently also on tissue confirmed that: (1) steroid receptors involve other transcription factors to activate their target genes [25, 29]; (2) transcription factors recruit histone mark writing, reading, and erasing coregulators; and (3) the co-regulating transcription factors are involved in the tissue specificity of the responses [48, 83]. The details on how different transcription factors cooperate during the transactivation via enhancers and chromatin histone modifications are being elucidated further. FoxA1 seems to act upstream of GATA2 as a pioneer factor for the AR [84]. However, the same transcription factor can have different roles depending on the enhancers under study (reviewed for AR in prostate cancer in [25, 85]).

Despite near identity of the DBDs, there are large differences between AR and GR in mobility reflecting kinetics of chromatin binding as measured by fluorescence recovery after photobleaching. These differences of residence at the MMTV enhancer array are compound effects of DNA binding as well as differential effects of other binding transcription factors, histon modifiers, and chromatin remodeling complexes [86, 87].

## Should we target the GR and AR simultaneously in castration-resistant prostate cancer

While AR can be targeted in castration-resistant prostate cancer with ADT or anti-androgens, glucocorticoids can be used to relieve pain or suppress androgens. A first clinical observation relevant for this discussion is that AR-targeting therapies can lead to the appearance of mutations that change ligand specificity of the AR and allows glucocorticoids to act as agonists for the AR [88].

In addition, the high similarity between the DBDs of AR and GR as well as their response elements led to the hypothesis that the receptors could be interchangeable [89]. In normal prostate epithelial cells, AR but not GR is expressed, so this is not relevant. During ADT treatment, however, GR can be upregulated and thus be co-expressed with the AR [11]. It was postulated, therefore, that in ADT or under anti-androgen therapy, the GR might take over the role of (inhibited) AR and thus lead to castration resistance or resistance against the latest AR-targeting therapies [10]. Clinically, this is an important question, since inhibitors of androgen synthesis, which are used to treat castration-resistant prostate cancer, are usually supplemented with glucocorticoids or glucocorticoid precursors to prevent side effects [90].

In prostate cancer cell lines, many genes that are androgen responsive can also respond to glucocorticoids, provided that the GR is expressed [41]. In such cells, the responses to androgens and glucocorticoids largely overlap, and overexpression of GR is correlated with a loss of anti-androgen control of proliferation [10]. Jaaskelainen et al. and Isikbay et al. showed that GR and AR both can activate anti-apoptotic genes, so when AR is inactivated by anti-androgens, the GR can still upregulate these genes [11, 91]. However, this seems in discordance with clinical practice, where glucocorticoids have been used in PCa treatment for many years. Their suppressive effects on serum androgen levels, their clinical benefit on PSA levels, and negative effects on tumor volume have been well established [92, 93]. Moreover, their inhibitory role on prostate cancer cell proliferation as well as angiogenesis has been documented in preclinical models [94, 95]. Therefore, while the possibility of GR taking over the survival role of the AR has been shown in preclinical work, at the moment, there is little evidence that this would be a major problem in the clinic. Nevertheless, there is need for well-controlled clinical studies to verify whether glucocorticoids could have adverse effects, maybe on a specific subtype of castration-resistant prostate cancer, which express GR. In the meantime, studying the role of AR and GR and their functional interactions in prostate cancer remains of major importance when developing and implementing new anti-androgen therapies [96].

## Future prospects

Over the last years, major steps forward have been taken in our knowledge of the transcription activation process. Surprisingly, we are still far from understanding the tissue-specific actions of AR and GR, two very similar steroid receptors with very divergent biological roles. Indeed, although general roadmaps of the activation are well described, now, we need to search for the details that explain the different rules of engagement of these two receptors.

The description of structures of full size receptors or larger receptor fragments bound to DNA, ligand, and coregulator peptides continues to reveal more details on the allosteric pathways within the receptors [44, 97]. The maturing of a number of technologies, such as ChIP-exo to better define the DNA-binding events [36], RIME to identify protein–protein interactions at the level of chromatin [98], and CRISPR/Cas9 to mutate enhancers or to reactivate genes expressing specific transcription factors or coregulators, now allows for the testing of the hypotheses on the allosteric signals of specific DNA elements on receptor functioning.
